# Forensic evaluation of the AmpF*l*STR^®^ NGM^™^ loci in Lodz region of Poland population sample

**DOI:** 10.1007/s00414-013-0882-z

**Published:** 2013-06-13

**Authors:** Maciej Jedrzejczyk, Renata Jacewicz, Jaroslaw Berent

**Affiliations:** Department of Forensic Medicine, Medical University of Lodz, Sedziowska 18a, 91-304 Lodz, Poland

**Keywords:** STR, Forensic genetics, Central Poland, Population

## Abstract

The paper is focused on population data for 15 polymorphic STR loci included in the NGM^TM^ amplification kit, obtained from a sample of 800 individuals from the Lodz region of Poland. Main statistical parameters of forensic interest were calculated and Hardy–Weinberg equilibrium was verified for each locus. Departure from HWE was not significant after applying Bonferroni corrected significance level for multiple testing (*p* = 0.0033). Comparative analysis between chosen populations was performed and some significant differences were found among investigated populations. Obtained values of parameters for NGM™ multiplex amplification kit point to wide range of possible applications of investigated STR markers to forensic genetics.

**Electronic supplementary material:**

The online version of this article (doi:10.1007/s00414-013-0882-z) contains supplementary material, which is available to authorized users.

The AmpF*l*STR^®^ NGM™ PCR kit is a high discriminatory short tandem repeat(STR) multiplex kit that combines 10 SGM Plus™ loci together with the five new EDNAP/ENFSI recommended loci (mini-STRs: D10S1248, D22S1045, D2S441; midi-STRs: D1S1656, D12S391), that were approved by the European Union Council for the expansion of the European Standard Set (ESS) of loci [1, 2]. The addition of new loci into ESS decreases the chance of obtaining false-positive matches with cross-border DNA data exchanges—especially when there are incomplete genetic profiles. The small amplicon sizes of the new loci increases the chance of amplification in degraded sample, what may be crucial in solving forensic cases where DNA may be fragmented and/or in low quantity [3]. There is lack of NGM™ loci population data from regions of Poland (except for Cracow and partially northern Poland populations) and that was the reason to establish a data set from a new region of Poland. Lodz, as a central region of Poland, can be regarded as a representative Polish population sample. Genetic polymorphism of 15 STR included in the NGM™ system was investigated in a sample of 800 unrelated, healthy, adult individuals from the Lodz region of central Poland (385 females and 415 males) involved in paternity testing, after consent. Genomic DNA was manually extracted from buccal swabs with the Swab Kit (A&A Biotechnology, Poland) according to manufacturer’s protocol. The concentration of extracted DNA was determined with the Qubit™ Quantitation Platform (Invitrogen, USA) and 7500 Real-Time PCR System with HID Real-Time PCR Analysis Software v.1.0 (Applied Biosystems, USA). Multiplex-PCR reaction was performed according to manufacturer of the AmpF*l*STR^®^ NGM™ PCR kit with approximately 0.5–1 ng of template DNA in a GeneAmp 9700 PCR System (Applied Biosystems, USA). PCR products were detected in a five-dye detection system and separated by capillary electrophoresis in a 3500 Genetic Analyzer (Applied Biosystems, USA). The results were analyzed with GeneMapper^®^ ID-X Software v.1.2 (Applied Biosystems, USA) using LIZ 600 v.2 size standard with reference allelic ladder and male 007 DNA control template provided with NGM™ kit. Allele designations were determined automatically by comparison of the amplified fragments with allelic ladder. Allele frequencies of autosomal loci are presented in ESM Table 2. Relevant population genetics statistical parameters such as polymorphism information content (PIC), power of discrimination, power of exclusion (PE), matching probability (MP), and typical paternity index (TPI) were calculated using PowerStats spreadsheet v. 1.2 [4]. Statistical analysis performed with GDA software included: expected heterozygosity (*H*_exp_), observed heterozygosity (*H*_obs_), and Hardy–Weinberg equilibrium (HWE) and linkage disequilibrium [5]. Possible deviations from expectations of HWE were tested by the exact test.

No significant deviations from Hardy–Weinberg equilibrium were found for 13 STR loci out of 15 NGM loci except for D2S441 and D3S1358 loci (*p* = 0.024 and *p* = 0.009, respectively) applying a *p* = 0.05 significance level. Detected weak deviations in those loci were found insignificant after applying Bonferroni correction for multiple testing (significance level at *p* = 0.0033) and all investigated loci met Hardy–Weinberg equilibrium expectations. No significant linkage disequilibrium between all pairs of loci was found after applying Bonferroni’s correction for multiple testing. Among five new markers included in NGM™ kit, for D1S1656 and D12S391 loci were found very high values of power of discrimination (0.980 and 0.974, respectively) and observed heterozygosity (0.888 and 0.868, respectively). The highest PIC value was noted for D1S1665 locus. Observed heterozygosity values among investigated 15 STR NGM™ markers ranged from 0.739 (D2S441) to 0.900 (D1S1656). Detailed information is presented in ESM Table 1. D1S1656 locus was found as an excellent marker included in NGM™ kit as it provided high level of genetic information needed in forensic analyses. The investigated marker set is characterized by very high value of forensically relevant statistics parameters, such as PE and MP. The combined PE and MP for all 15 loci are 0.9999998 and 1.61 × 10^−19^, respectively.

Alleles and frequencies of NGM™ loci observed in population from region of Lodz (*n* = 800) were compared with selected, previously published, data available for different world’s populations (ESM Table 1). Observed distributions of the alleles frequencies among Lodz population were similar to other European populations. Among Polish populations, performed comparison revealed differences between Lodz and Cracow samples in D21S11 and D12S391 loci but were found insignificant after applying Bonferonni’s correction (*p* < 0.001). However, statistically significant differences were noticed for the rest of investigated populations (several differences) and the difference between Lodz and Chinese population appeared to be the most visible with disconcordance within every locus. Revealed differences between analyzed Polish and world’s populations strongly substantiate the need of creating such databases.

The concordance between Identifiler^®^ and NGM™ amplification kit was also investigated by choosing randomly 20 samples from paternity cases and 20 samples from forensic criminal cases (ESM Table 3). Genotyping of paternity samples resulted in obtaining the same genetic profiles for both STR kits. Moreover, we noticed that levels of RFU were much higher in samples amplified with NGM™ than in samples amplified with Identifiler^®^ kit (ESM Figs. 1 and 2). Our findings on NGM™ kit also resulted in obtaining very good efficiency and complete 15-STR profiles with chosen forensic samples that were previously incomplete typed with Identifiler^®^ kit (ESM Figs. 3 and 4). As little as 0.1 ng of DNA is sufficient to get a full loci profile using NGM™ system what is about 10 % less than the amount of DNA required for Identifiler^®^.

Performed experiment leads to a conclusion that NGM™ kit has very high level of sensitivity and is an extremely discriminatory multiplex-STR system, what is very important for international cross-countries data sharing and analyzing challenging casework samples. NGM™ is one of the most genetically informative STR kits available for forensic purposes, making a remarkable improvement in forensic genetics routine analyses and providing supplementary genetic information.

Laboratory participates in GEDNAP and Polish Society for Forensic Genetics and Criminology (www.ptmsik.pl) proficiency tests for forensic DNA typing certificates.

This paper follows the guidelines for publication of population data requested by the journal [6].

## Electronic supplementary material

Below is the link to the electronic supplementary material.

ESM 1 (XLSX 16 kb)

ESM 2 (XLS 33 kb)

ESM 3 (XLS 49 kb)

ESM 4 (DOC 468 kb)

ESM 5 (DOC 526 kb)

ESM 6 (DOC 526 kb)

ESM 7 (DOC 547 kb)
