# Supplementary material for: Forensic evaluation of the AmpFlSTR® NGM™ loci in Lodz region of Poland population sample
Source: Int J Legal Med. 2013 Jun 13;127(5):911–2. doi: 10.1007/s00414-013-0882-z (PMC3751388; doi:10.1007/s00414-013-0882-z)

Fig. 2. The example of a paternity sample (P04) genetic profile obtained with NGM kit. Peak height is indicated below allele designation.


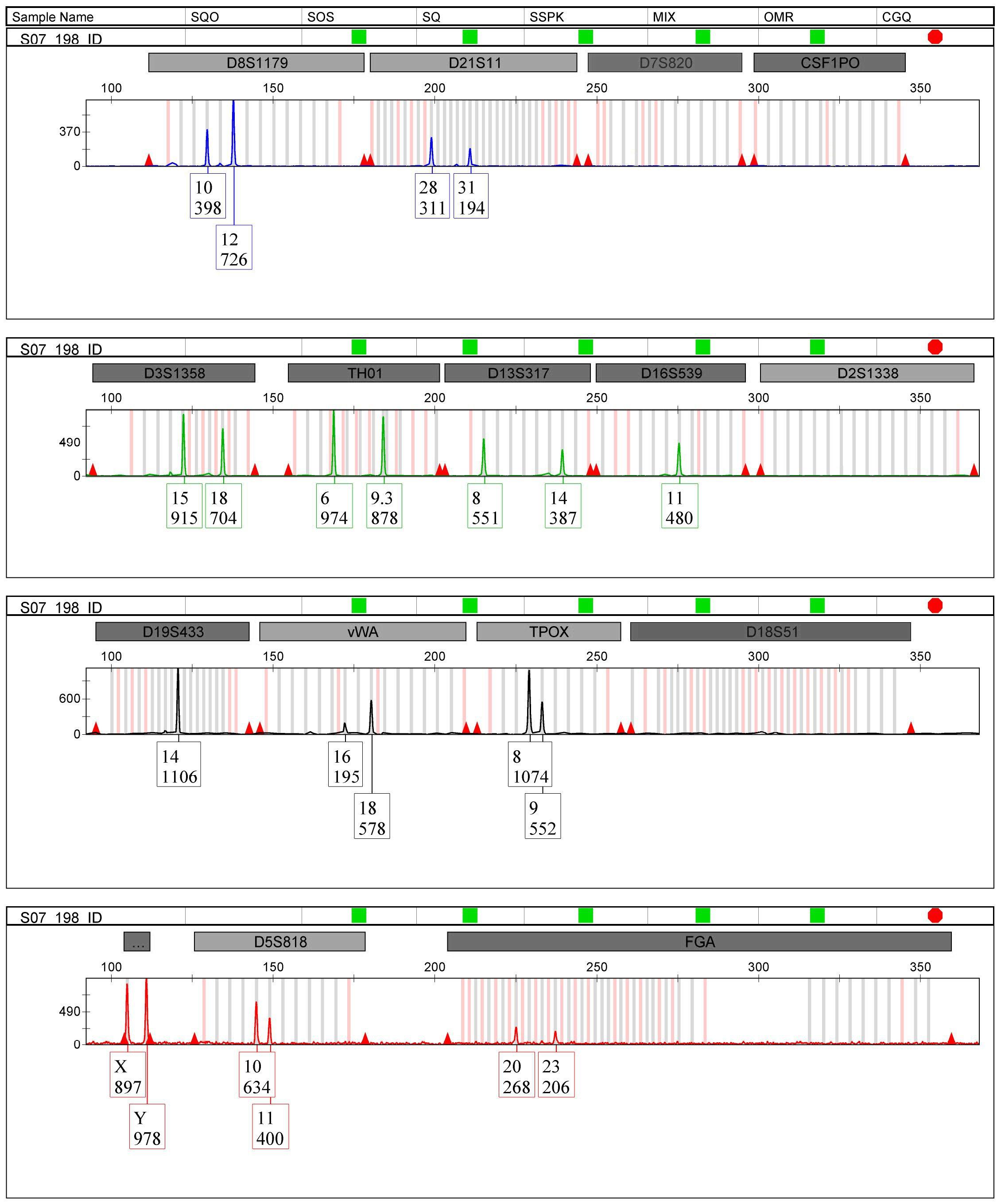

Supplement: Supplementary file 5 — (DOC 526 kb) [file 414_2013_882_MOESM5_ESM.doc]
