# Supplementary material for: Forensic evaluation of the AmpFlSTR® NGM™ loci in Lodz region of Poland population sample
Source: Int J Legal Med. 2013 Jun 13;127(5):911–2. doi: 10.1007/s00414-013-0882-z (PMC3751388; doi:10.1007/s00414-013-0882-z)
Supplement: Supplementary file 6 — (DOC 526 kb) [file 414_2013_882_MOESM6_ESM.doc]

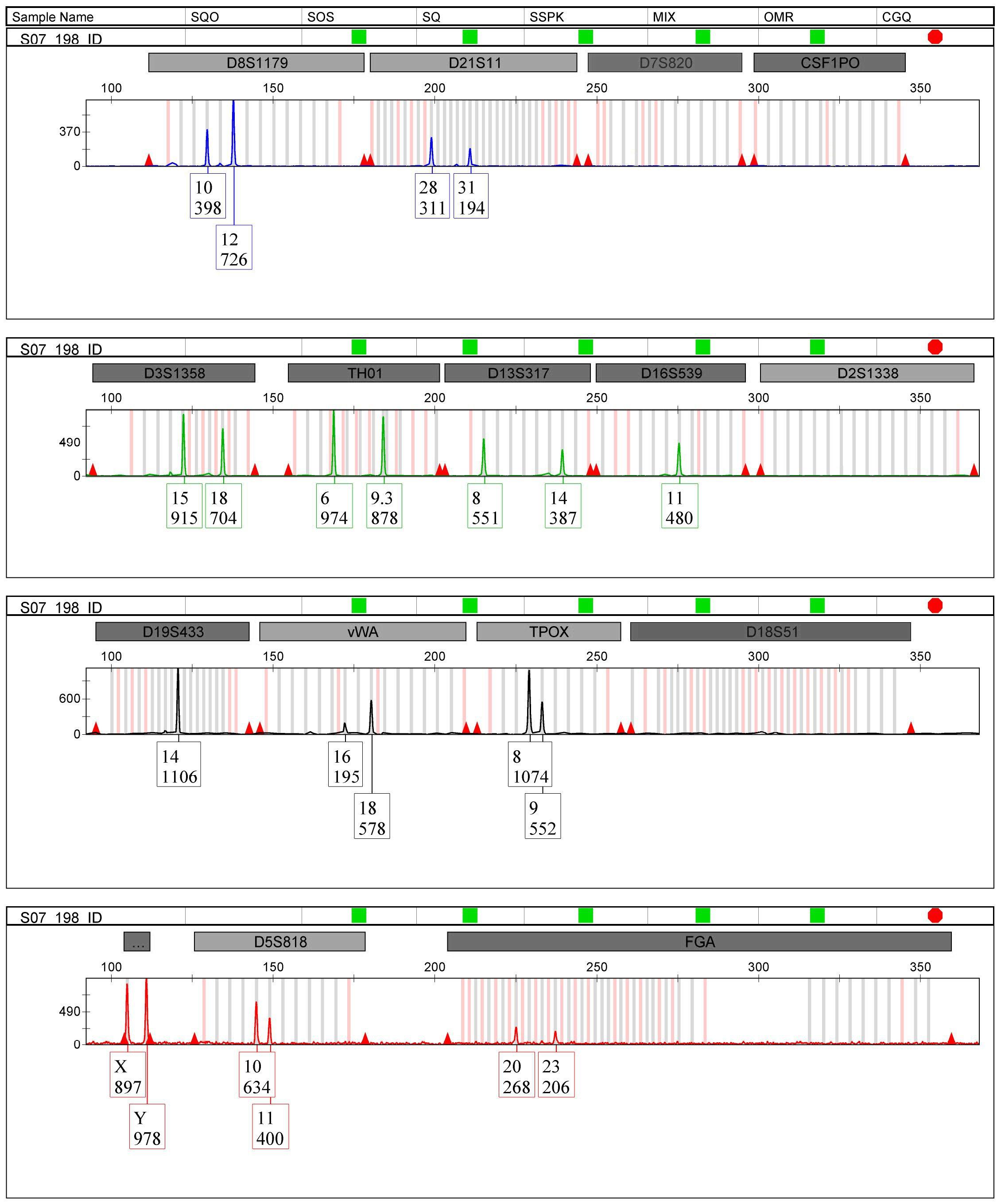


Fig. 3. The example of a forensic sample (S07) genetic profile obtained with Identifiler kit. Peak height is indicated below allele designation.
